# Pharmacokinetic, Hemostatic, and Anticancer Properties of a Low-Anticoagulant Bovine Heparin

**DOI:** 10.1055/a-1750-1300

**Published:** 2022-07-11

**Authors:** Roberto P. Santos, Ana M.F. Tovar, Marcos R. Oliveira, Adriana A. Piquet, Nina V. Capillé, Stephan N.M.C.G. Oliveira, Ana H. Correia, José N. Farias, Eduardo Vilanova, Paulo A.S. Mourão

**Affiliations:** 1 Hospital Universitário Clementino Fraga Filho and Instituto de Bioquímica Médica Leopoldo de Meis, Laboratório de Tecido Conjuntivo, Hospital Universitário Clementino Fraga Filho and Instituto de Bioquímica Médica Leopoldo de Meis, Universidade Federal do Rio de Janeiro, Rio de Janeiro, Brazil; 2 Hospital Universitário Clementino Fraga Filho, Serviço de Anatomia Patológica, Hospital Universitário Clementino Fraga Filho, Rio de Janeiro, Brazil; 3 Hospital Universitário Clementino Fraga Filho, Laboratório Multidisciplinar de Pesquisa, Hospital Universitário Clementino Fraga Filho, Rio de Janeiro, Brazil

**Keywords:** unfractionated heparin, low-molecular weight heparin, pharmacokinetics, bleeding, Lewis lung carcinoma

## Abstract

Heparin is a centennial anticoagulant drug broadly employed for treatment and prophylaxis of thromboembolic conditions. Although unfractionated heparin (UFH) has already been shown to have remarkable pharmacological potential for treating a variety of diseases unrelated with thromboembolism, including cancer, atherosclerosis, inflammation, and virus infections, its high anticoagulant potency makes the doses necessary to exert non-hemostatic effects unsafe due to an elevated bleeding risk. Our group recently developed a new low-anticoagulant bovine heparin (LABH) bearing the same disaccharide building blocks of the UFH gold standard sourced from porcine mucosa (HPI) but with anticoagulant potency approximately 85% lower (approximately 25 and 180 Heparin International Units [IU]/mg). In the present work, we investigated the pharmacokinetics profile, bleeding potential, and anticancer properties of LABH administered subcutaneous into mice. LABH showed pharmacokinetics profile similar to HPI but different from the low-molecular weight heparin (LMWH) enoxaparin and diminished bleeding potential, even at high doses. Subcutaneous treatment with LABH delays the early progression of Lewis lung carcinoma, improves survival, and brings beneficial health outcomes to the mice, without the advent of adverse effects (hemorrhage/mortality) seen in the animals treated with HPI. These results demonstrate that LABH is a promising candidate for prospecting new therapeutic uses for UFH.

## Introduction

Unfractionated heparin (UFH) obtained from animal tissues has been massively exploited as anticoagulant agent for almost a century, which makes it one of the oldest extant biologic drugs. 1 Although low-molecular weight heparins (LMWHs) and directly acting oral anticoagulants have been increasingly prescribed for treatment and prophylaxis of most thromboembolic diseases, UFH is still the most potent anticoagulant, being indispensable for patients requiring a rapid-onset and deep low-coagulant state, such as those undergoing cardiopulmonary bypass, extracorporeal membrane oxygenation, hemodialysis, and severe deep-vein thrombosis and pulmonary embolism. 2 In addition to anticoagulant activity, UFH also exerts many other biological effects, including modulation of different proteases and components of the extracellular matrix and binding to cytokines and growth factors. 3

Several preclinical and clinical studies have already demonstrated that UFH has a remarkable pharmacological potential for treating diseases unrelated to thromboembolism such as cancer, atherosclerosis, inflammation, and viral infections. 4 5 Among them, we can highlight the therapeutic effects of UFH on different pathways related to cancer progression, including angiogenesis, tumor cell proliferation and adhesion, immune system modulation and tumor cell migration and invasion during metastasis. 6

However, the high anticoagulant potency of UFH makes the doses commonly necessary to achieve satisfactory effects on therapeutic targets related to cancer or other non-thromboembolic diseases unsafe due to an elevated risk of hemorrhage incidents. 7 In addition to this serious adverse effect, UFH is clinically employed by intravenous route, making it unfeasible for long-term outpatient treatments. 8 Although LMWHs and some UFH mimetics obtained through extensive chemical/enzymatic processes have already proven to be effective and pose reduced risk of bleeding, different stakeholders involved in the production and research of heparins are still looking for new and more feasible UFH derivatives that somehow preserve the physical–chemical features required for aiming new therapeutic targets but with decreased anticoagulant activity nonetheless. 9

**Fig. 1:**
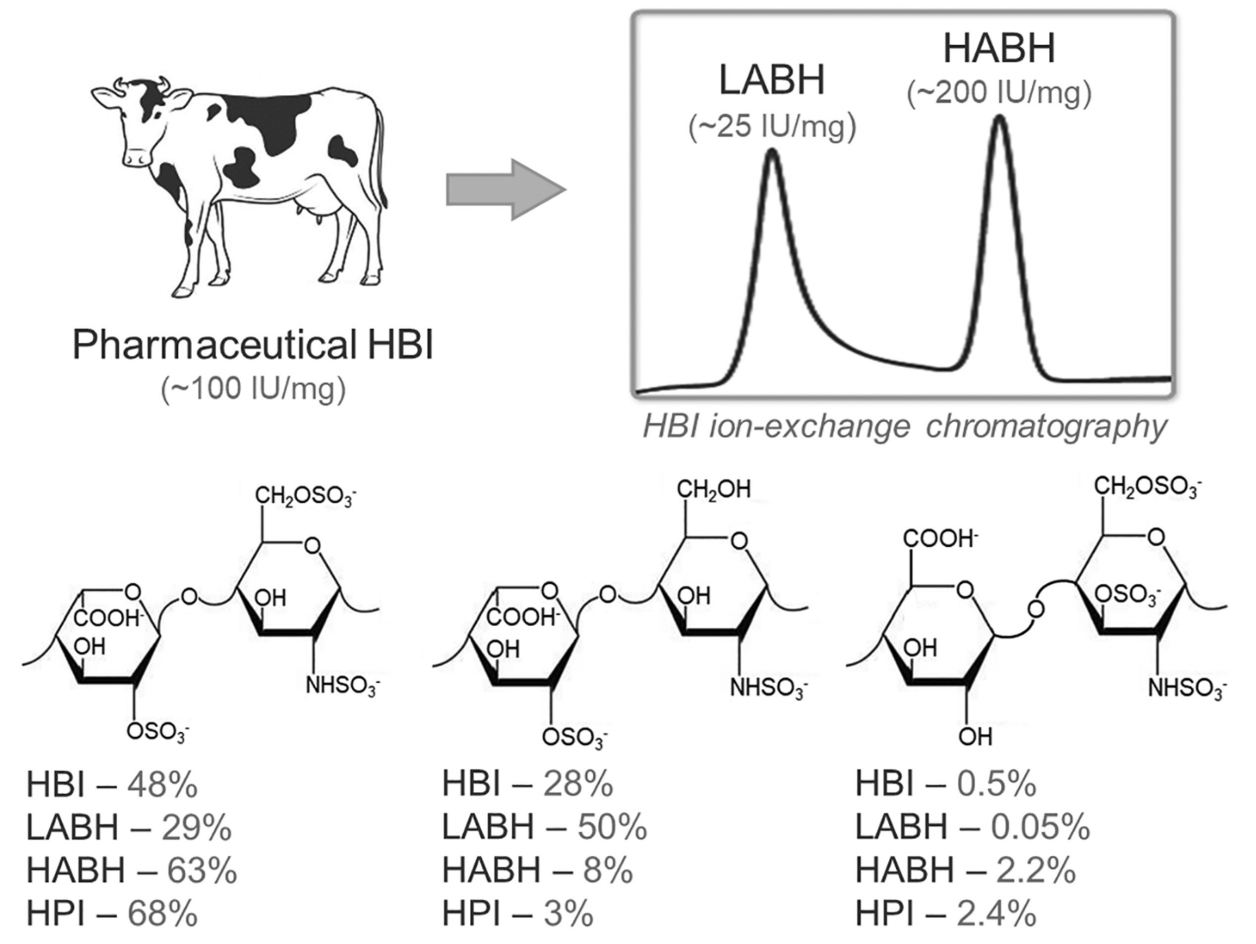
Novel bovine heparins. Average pharmaceutical bovine heparin preparations (HBI) with approximately 100 IU/mg anticoagulant potency are actually composed by a mixture of low-anticoagulant (approximately 25 IU/mg) and high-anticoagulant (200 IU/mg) heparin chains named LABH and HABH, respectively, which can be separated through a single anion-exchange chromatography step. These bovine derivatives, as well as native HBI and porcine heparin (HPI), are composed by the same disaccharide units but in different proportions. HABH has a disaccharide composition similar to HPI while LABH contains lower proportions of *N* ,6-disulfated and *N* ,3,6-trisulfated α-glucosamine units, which are important components of AT-link region of heparin. For further details on structural and pharmacological features of these heparins see Tovar et al 2019. 18

All UFHs currently available for clinical use and production of LMWHs are produced using heparin porcine intestine (Heparin Porcine Intestine; HPI) (Heparin Bovine Intestine; HBI) Hepatocyte Growth Factor (HGF) as raw material, except in some countries, including Brazil, Argentina, and India, which also employ UFH products obtained from heparin bovine intestine (HBI). 10 11 12 13 However, the use of HPI and HBI as interchangeable UFHs requires special attention due to their chemical and pharmacological differences. 14 The increased proportion of *N* -sulfated but not 6-sulfated α-glucosamine and diminished quantity of the disaccharide composed of *N* ,3,6-trisulfated α-glucosamine linked to β-glucuronic acid ( Fig. 1 ), which is directly involved in the potentiation of antithrombin (AT), makes the anticoagulant activity of HBI significantly lower than that of HPI (approximately 120 vs. 180 international unit [IU]/mg, respectively). 15 16 17 Nevertheless, our research group demonstrated that pharmaceutical HBI is actually composed of a mixture of two types of heparin chains bearing different chemical compositions and anticoagulant activities, which in turn can be separated through a single ion-exchange chromatography step ( Fig. 1 ), named as high-anticoagulant bovine heparin (HABH) and LABH. 18 19 While HABH has chemical composition (enriched in *N* ,6-disulfated α-glucosamine) and anticoagulant activity (approximately 200 IU/mg) similar to HPI, LABH has diminished potency (approximately 25 IU/mg) due to the preponderance of disaccharides containing *N* -sulfated but not 6-sulfated α-glucosamine ( Fig. 1 ). Different from HBI, HPI and pharmaceutical preparations sourced from bovine lung contain diminished amounts of low-anticoagulant heparin chains such as the component of LABH. 18

Considering that LABH has anticoagulant potency markedly lower than that of the gold standard HPI and consequently reduced bleeding risk, it is a suitable candidate for prospecting novel pharmaceutical uses of UFH. 20 In the present work, we evaluate the pharmacokinetics profile and bleeding potential of LABH administered subcutaneous (SC) through animal models. We also find that SC treatment with LABH delays the early Lung Lewis Carcinoma (LLC) tumor progression in mice and improves survival and brings beneficial health outcomes (reduced weight loss and incidence of complicated tumors) to the sick animals. Besides the potential for development as a new oncologic coadjutant, the establishment of basic pharmacological parameters, such as pharmacokinetics and safety, is paramount for further researches on the use of LABH as a therapeutic agent for treatment of other non-thromboembolic diseases.

## Material and Methods

### Heparins

LABH employed in the assays was prepared by fractionating a pool containing 10 batches of pharmaceutical preparations of HBI available in the Brazilian market through ion-exchange chromatography, by following the “Protocol 1” described in Tovar et al. 18 Pharmaceutical HPI (Hemofol) was obtained from Cristália (Itapira, Brazil) and the LMWH enoxaparin (Clexane) from Sanofi (Singapore). The Sixth International Heparin Standard (2,145 units per vial, Lot No. 07/328) and the Third International Standard for LMWH (Lot 11/176) were obtained from the National Institute for Biological Standards and Control (NIBSC; Potters Bar, United Kingdom).

### Animal Experiments

Experiments were conducted with adult (8–13 weeks age) wild type C57Bl/6 mice maintained at 22 to 24°C, artificial light cycles of 12 hours, and *ad libitum* feeding. The animals submitted to invasive/surgical procedures were anesthetized with 35 mg/kg ketamine and 9 mg/kg xylazine (both from Ceva Brasil; Paulínia, Brazil) administered intraperitoneally. All in vivo assays were performed in compliance with the guidelines for animal care and experimentation of our institution (Federal University of Rio de Janeiro, Brazil).

### In Vitro Anti-FIIa and -FXa Activities

The anticoagulant activities of the heparins were determined by measuring inhibition of thrombin (FIIa) and activated factor X (FXa) with chromogenic assays, as previously described. 21 Different concentrations of HPI, LABH, and LMWH (0→0.4 μg/mL) were incubated (60 seconds at 37°C) with 10 nM AT and 2 nM FIIa or FXa (Hematologic Technologies; Essex Junction, United States) or human plasma. After incubation, the anti-FIIa or -FXa activities were determined by adding 100 μM of chromogenic substrates S-2238 or S-2765 (Chromogenix; Molndal, Sweden), respectively, and then recording absorbance (405 nm) during 300 seconds in a ThermoMax Microplate Reader (American Devices; Sunnyvale, United States). Anti-FIIa and -FXa potencies (IU/mg) were calculated with basis on parallel line assays performed with the Sixth International Heparin Standard (for LABH and HPI) and with the Third Standard for LMHW.

### Pharmacokinetic Assessments

Plasmatic concentrations of the heparins were indirectly estimated by measuring residual anti-FXa or anti-FIIa activity, as described elsewhere. 22 Briefly, animals treated with doses of 2, 8, and 20 mg/kg of LMWH, HPI, and LABH, respectively, were administered SC. Blood samples were collected by the inferior vena cava at different times after the treatment (0 → 10 hours). The anti-FXa or anti-FIIa activities (IU/mL) were measured as described in the previous section by using different dilutions of the plasmas from each treatment and then calculated on the basis of the values obtained to naïve plasma spiked with known concentrations of HPI, LABH, or LMWH.

### Bleeding Evaluations

Bleeding tendencies of the heparins were assessed on the basis of in vivo model described in Tovar et al. 18 Briefly, animals were treated SC with different doses of LABH (8→40 mg/kg), HPI (1→16 mg/kg), LMWH (8→16 mg/kg) and saline (control). Bleeding was quantified after 1 hour of heparin administration by collecting the blood spilled from cuts (1 mm diameter) in the tails of the animals in 1.5 mL distilled water. Blood was collected for 10 minutes and then in the subsequent 50 minutes and hemorrhage quantified by measuring the dissolved hemoglobin (absorbance 540 nm) with a ThermoMax Microplate Reader.

### Lewis Lung Carcinoma Model in Mice

LLC cells obtained from ATCC (Manassas, United States) were grown in modified Dulbecco-Eagle (DMEM) medium (Vitrocell; Campinas, Brazil), supplemented with 10% fetal bovine serum (Invitrogen; Waltham, United States). The LLC cells (5 × 10 ^5^ cells/60 µL) were inoculated SC into the dorsal region of the mice. One day after the inoculation, the animals were treated with SC injections of LABH, HPI, or LMWH (8 mg/kg of each) or saline (control) once daily during 26 days (D27). Growing of the tumors was monitored weekly (D1, D7, D14, D21 and D28) by measuring their cranium-caudal and lateral–lateral axes. After the treatment period (D28), the animals were sacrificed and then the tumors were surgically resected and weighed.

### Evaluation of Health Parameters

Different health parameters of both heath (naïve) and sick animals treated with different heparins or saline (control) were evaluated. Body weight of the animals was monitored weekly (D1, D7, D14, D21, and D28) during the treatment period. Weighing of the animals on D28 was subtracted from the tumor weights. Ectoscopic evaluations were based on the onset and severity of ulcerations and hematomas caused by the tumors or application of the heparins during or after (post-mortem) treatment period. Mortality rate of the animals submitted to different treatments was recorded daily and the cause of death was assessed by macro-pathological necropsy, ulceration, and/or critical health conditions were criteria to sacrifice the animal. The lungs of the animals were evaluated for the occurrence of metastasis by histological examination. A minimum of three slides of each animal, stained with hematoxylin-eosin were carefully examined.

## Results and Discussion

### LABH Differs from HPI and LMWH on the Profile of Anticoagulant Activity

**Fig. 2:**
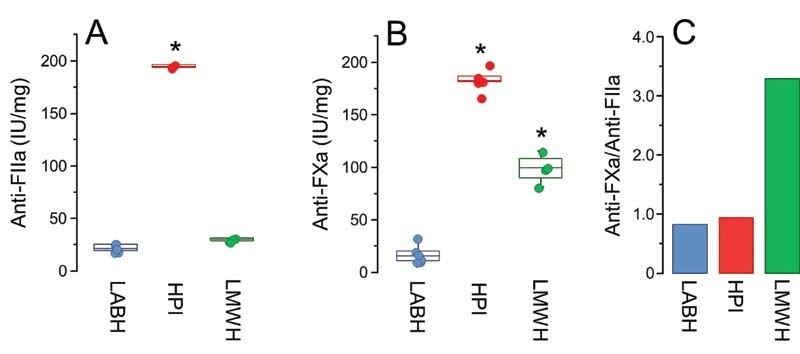
Anti-FIIa ( **A** ), anti-FXa ( **B** ) activities and anti-FXa/anti-FIIa ratios ( **C** ) of LABH, HPI, and the LMWH enoxaparin. Results expressed as IU/mg were calculated with basis on parallel line assays performed with the 6 ^th^ International Heparin Standard for LABH and HPI and with the 3 ^rd^ Standard for LMWH. * indicates *p* < 0.05 in relation to LABH (ANOVA with Bonferroni post hoc test).

We evaluated the in vitro anticoagulant activity of LABH based on the anti-FIIa and anti-FXa activities and compared the effect with those of HPI and LMWH. Clearly, the three types of heparins present different activity profiles. LABH had low and HPI high anticoagulant activities in both assays (approximately 25 IU/mg and approximately 180 IU/mg, respectively; Fig. 2 ). In contrast, LMWH (enoxaparin) showed potent anti-FXa but low anti-FIIa activities (approximately 100 IU/mg and approximately 25 IU/mg; Fig. 2 ), as previously established elsewhere. 22 23 24 Although its low potency had already been determined by Tovar et al, 18 the distinct profile of LABH seen in these anticoagulant assays indicates that this heparin may also have different therapeutic effects than HPI and LMWH on non-hemostatic pathological conditions. We also found that the anti-FXa/anti-FIIa ratios of LABH in assays conducted with purified AT or human plasma (containing both AT and heparin co-factor II [HCII]) are approximately 1 (data not shown). Considering that HCII mediates anti-FIIa but not anti-FXa activity, this indicates that the contribution of HCII for the anticoagulant activity of LABH is similar to those previously reported in both HPI and HBI 18

### LABH and HPI Administered SC Have Similar Pharmacokinetics Profile

**Fig. 3:**
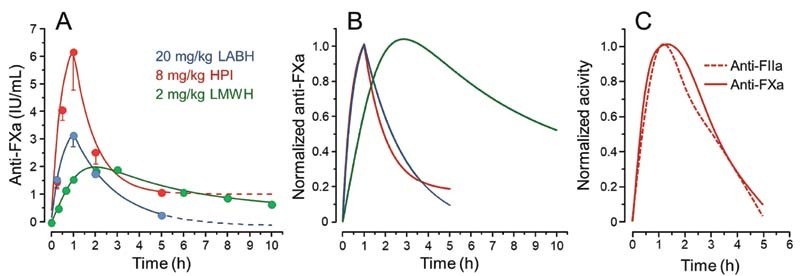
Pharmacokinetics profiles of HABH, HPI, and LMWH. Doses of 2, 8, and 20 mg/kg body weight of LMWH, HPI, and LABH, respectively, were SC administered to mice. Blood samples were collected by the inferior vena cava at different periods of time and heparin concentrations in the plasma were determined by the anti-FXa assay. Panel ( **A** ) shows the effective results and panel ( **B** ) the data for each heparin normalized to a maximum value of 1 based on plasma anti-FXa activities and the panel ( **C** ) the comparison of normalized profiles of HPI monitored by anti-FXa ( *solid red line* ) or anti-FIIa ( *dashed red line* ) activities.

The following step on the way to propose a therapeutic use for LABH is to assure its absorption after SC administration and define its pharmacokinetic profile. To achieve this objective, LABH, HPI, and LMWH were administered SC to mice at doses adjusted to assure their detections in the plasma by anti-FXa assay. The low anticoagulant activity of LABH requires administration of a higher dose (20 mg/kg) to make feasible its detection in the mice plasma. LABH and HPI have similar pharmacokinetics profiles ( Fig. 3 ), as estimated by the time required to achieve their maximum plasma concentrations (T _max_ ) and the half-life time (T _1/2_ ), which are markedly distinct from the profile of LMWH ( Table 1 ). Clearly, we observed a similarity between the absorption of LABH and HPI but not with LMWH.

**Table 1:** Pharmacokinetic parameters T _max_ (time to reach maximum plasma concentration), T _1/2_ (elimination half-life), AUC _0–10__h_ (area under the curve) and C _max_ (maximum plasma concentration) and molecular masses ( *M _w_* ) of LABH, HPI, and LMWH

	*T*_max_ (h)	*T*_1/2_ (h)	AUC _0–10h_ (µg/mL) a	*C*_max_ (µg/mL) a	* M _w_* (Da) b
LABH	1	0.84 ± 0.3	42.0	16.5	approximately 13,500
HPI	1	1.52 ± 0.8	25.5	8.5	approximately 17,000
LMWH	2	4.89 ± 0.2	126.0	19.9	approximately 4,500

Abbreviations: LABH, low-anticoagulant bovine heparin; LMWH, low-molecular weight heparin.

a Normalized values in µg of heparin per mL of plasma adjusted for doses of 2 mg/kg of LABH and HPI and specific potency (IU/mg) of each heparin.

b Data from Tovar et al 2019 18 and Oliveira et al 2015. 22

When the maximum plasma concentrations (C _max_ ) and also the area under the curve (AUC) were normalized to a similar dose and to their specific anti-FXa potencies, we observed that LABH is better absorbed than HPI but poorer than LMWH ( Table 1 ). Although differing in the proportion of some disaccharide building blocks, the improved absorption of LABH in comparison to HPI certainly relates to its smaller molecular weight (13.5 and 17 kDa, respectively), which confirms the inverse correlation between SC suitability and molecular weights of the heparins. 25 We also observed that the pharmacokinetics profiles of HPI administered SC by monitoring anti-FXa or anti-FIIa activities are similar ( Fig. 3C ). Although there is little information on the pharmacokinetics of UFHs after SC administration, our results are in line with the literature reporting that high-molecular-mass heparins have poor SC pharmacokinetics and are eliminated by both renal filtration and endothelium cells capitation while LMWHs are better absorbed and removed from plasma mostly by renal route. 26

### LABH-Administered SC Does Not Provoke Bleeding

**Fig. 4:**
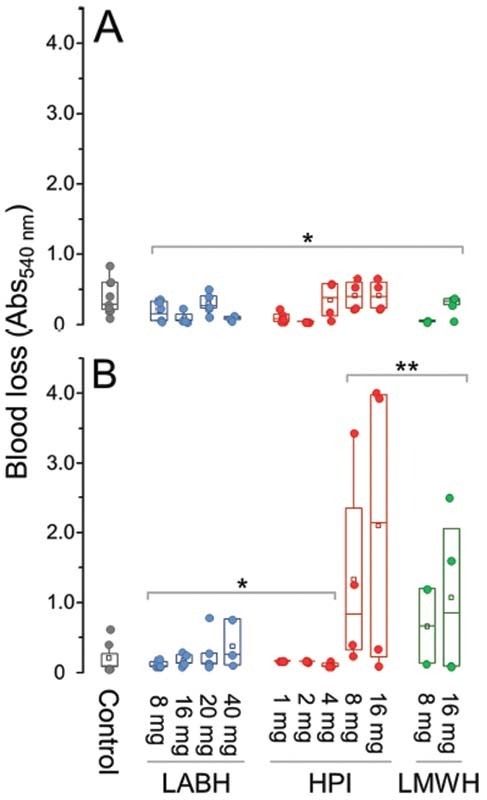
Bleeding evaluations of the three types of heparins. Different doses of LABH, HPI, LMWH, and saline (control) were administered SC to mice. Bleeding was quantified by collecting the blood spilled from cuts in the tail of the animals in distilled water for an initial period of 10 minutes ( **A** ) and in the subsequent 50 minutes ( **B** ). The dissolved hemoglobin was determined by absorbance at 540 nm. * indicates *p* > 0.05 and ** *p* < 0.05 in relation to the control (ANOVA with Bonferroni post hoc test).

Next, we evaluated the bleeding effect of LABH after SC administration. Bleeding is the major adverse effect of heparin, especially in the case of UFHs. 27 28 It is an obstacle for the use of UFHs in non-thromboembolic diseases, especially because such uses often require elevated doses and long periods of administration. 29 We evaluated bleeding measuring the blood spilled from cuts in tails of mice 1 hour after SC administration of LABH, HPI, and LMWH. Blood was collected for an initial period of 10 minutes and then in the subsequent 50 minutes ( Fig. 4 ).

In the initial 10 minutes of blood collection, none of the heparins provoked blood losses significantly higher than those of the animals treated with saline (control) ( Fig. 4A ). Possibly, the mechanisms of primary hemostasis (e.g., vasoconstriction, change in vascular permeability and platelet adhesion) are able to assuage the initial bleeding, even in the animals heparinized with HPI. 30 31 In the subsequent 50 minutes, doses of HPI and LMWH above 8 mg/kg resulted in blood losses significantly higher than that measured in control animals ( Fig. 4B ). On the other hand, SC administration of LABH did not increase bleeding, except for a modest effect in the animals treated with a very high dose (40 mg/kg).

Tovar et al showed that different from HPI, HBI, and the bovine derivative HABH, LABH did not provoke bleeding even by intravenous (IV) administration. 18 Such a lack of hemorrhage effects directly correlates with the low anticoagulant potency of LABH (approximately 25 IU/mg). Although LMWHs are certainly safer than non-modified UFHs, 32 33 we observed that even high SC doses of LABH provoked less bleeding than enoxaparin in the animals, this is possibly due to the improved SC absorption and high anti-FXa potency (approximately 100 IU/mg) of this LMWH. In conclusion, LABH is devoid of bleeding effect by both SC and IV administration and thus do not pose the worse adverse effect hindering the therapeutic use of UFHs in non-thromboembolic diseases.

### Effect of LABH on LLC Tumor Progression

**Fig. 5:**
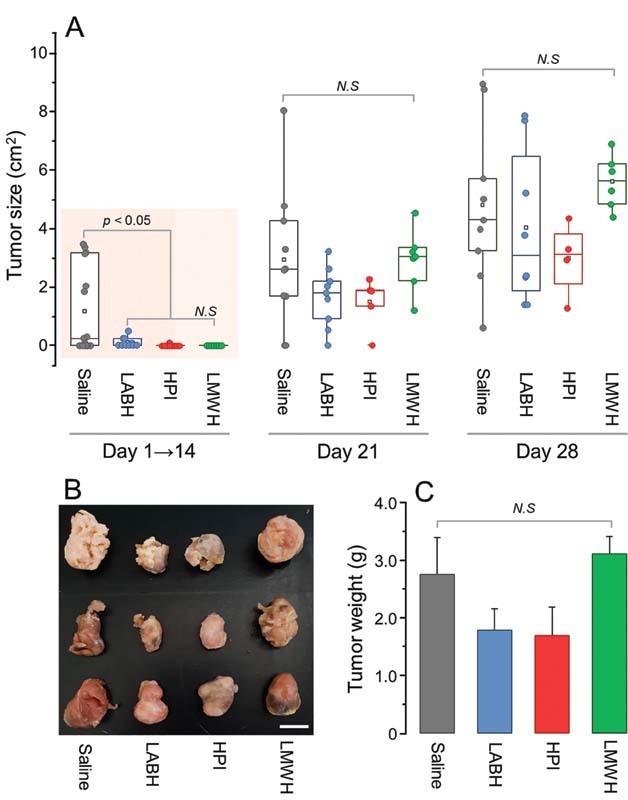
Progression of LLC tumors in mice treated with LABH, HPI, LMWH, and saline (control). LCC cells (5 × 10 ^5^ cells in 60 µL) were inoculated SC into the dorsal region of mice. ( **A** ) One day after inoculation, the mice received daily SC injections of the different heparins for 28 days. Growing of the tumors was monitored weekly measuring their cranium-caudal and lateral–lateral axes. On the 28 ^th^ day the animals were sacrificed and then the tumors were resected ( **B** ) and weighed ( **C** ). N.S., non-significant ( *p* > 0.05; ANOVA with Bonferroni post hoc test).

Anticancer effect is one of the most relevant non-hemostatic pharmacological activities reported for heparins. 34 35 36 We assessed this effect of LABH in comparison with those of HPI and LMWH using an experimental model of cancer based on tumor formation by SC inoculation of LLC cells in mice. 37 The animals received daily SC doses (8 mg/kg) of the three heparins. Measurement of the tumor size by examination of the animals showed that the three heparins delayed tumor progression, which is more evident up to the 14 ^th^ day and became less expressive on the examinations at the 21 ^st^ and especially at the 28 ^th^ days ( Fig. 5A ). Tumor was detected on the 14 ^th^ day in nine animals among 15 receiving saline and only in four among 10 treated with LABH, all with a small size (<0.5 cm ^2^ ). None of the seven animals treated with LMWH had tumor and just one among the same number of animals was treated with HPI (shadowed area in Fig. 5A ).

On the 28 ^th^ day after tumor cells inoculation, the animals were sacrificed and their tumors resected, examined, and weighted. Again, we observed that the heparins delayed tumor progression, which was less evident in the case of LMWH ( Fig. 5B, C ). Animals treated with LABH and HPI showed a decrease in tumor size, but with no statistical significance, in comparison with the control animals. We did not identify tumor metastasis on lungs of all animals' groups after careful histological examination (not shown).

**Fig. 6:**
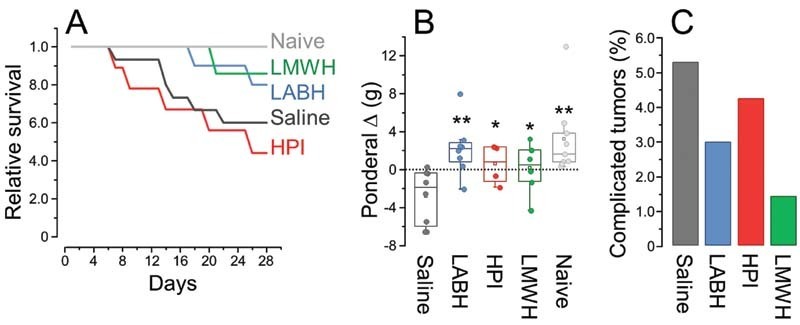
Relative survival ( **A** ), ponderal weight variation ( **B** ), and percentage of complicated tumors ( **C** ) of mice inoculated with LLC cells treated with the different heparins and saline (control) and naïve animals. * indicates *p* > 0.05 and ** *p* < 0.05 in relation to the control (ANOVA with Bonferroni post hoc test).

Subsequently we attempted to examine the balance between the beneficial action of the three heparins on the tumor progression and possible adverse effects of the drugs. The relative survival curves ( Fig. 6A ) showed that animals inoculated with LLC cells and treated with HPI or saline had a high mortality rate, most of them were related to ulcerations. Some animals treated with HPI that died prematurely showed extensive post-mortem dorsal hematoma at the injection site, in addition to pulmonary hemorrhages, suggesting that bleeding is the major cause of death or at least contributed to that. These findings did not occur in other groups of mice. The animals treated with LABH and LMWH showed a notable decrease in mortality compared with the animals treated with saline or HPI.

Another approach to examine the beneficial effects of the heparins during tumor progression was based on the ponderal loss/gain of body weight in the course of 28 days treatment. Mice inoculated with LLC cells and treated with LABH showed an average body weight increase of approximately 11%, similar to the naive group (approximately 14%) ( Fig. 6B ). Animals treated with saline (control) had a decrease of 9% in body weight while HPI and LMWH had more modest effect on the increase of the mice body weight (approximately 3 and 6%, respectively). We also evaluated the incidence of complicated tumors in the animals based on ectoscopic examination. The three types of heparins had favorable effects preventing tumor ulceration and hematomas but LABH and LMWH showed more pronounced benefices ( Fig. 6C ).

Although most of the studies on the anticancer properties of heparins are focused on their P-selectin-mediated hematogenous anti-metastatic activities, both UFHs and LMWHs have also proven to be effective for other cancer therapeutic targets, such as upregulation of E-cadherin and inhibition of HGF, heparinase, and galectin-3. 38 39 40 41 42 UFH inhibits proliferation of tumor cells by modulating the proto-oncogenes c-Myc and c-Fos, which downregulates the phosphorylation of MAPK part of the signaling cascade of protein kinase C, thus hindering the growth of both primary and metastatic tumors. 43 Another noteworthy anticancer effect of heparins is the release of the natural anticoagulant TFPI (tissue factor pathway inhibitor) from the endothelial cells. TFPI has been shown to inhibit hypercoagulability and angiogenesis resulting from the overexpression of tissue factor by tumor cells. 44 Besides these hemostatic effects, TFPI, especially TFPI-2 synthesized by vascular cells, have also been demonstrated to exert anti-metastatic activity by downregulating effectors involved in the degradation of the extracellular matrix during extravasation or intravasation, such as heparanase and matrix metalloproteinase-1. 45 46

Other heparin/heparinoid derivatives devoid of anticoagulant activity have already exhibited anticancer effects. A heparan sulfate hexasaccharide has proven to inhibit cancer stem cells renewal and induces apoptosis of three types of tumor cells. 47 Another study tested a low-anticoagulant heparin on patients with myeloid leukemia, aiming the survival of the leukemic stem cells in the marrow bone, which resulted in increased remission/recovery rates and no adverse events. 48 The sulfated non-anticoagulant heparin (S-NACH) increases threefold TFPI-2 levels and suppresses pancreatic tumor growth and metastasis in animal models. 49 50 Notwithstanding we have not demonstrated a specific mechanism of action, the delay in the early LLC tumor progression, improved survival and health (reduced weight loss and incidence of complicated tumors) promoted by SC administration of doses deprived of bleeding risk of the low-anticoagulant LABH in the mice must not relates to survival/viability of cancer stem cells, considering that it did not affect the final size of the tumors, but to a possible raise in the plasmatic levels of TFPI.

## Conclusion

Several clinical trials have shown that both UFH and LMWHs might bring beneficial clinical outcomes to patients with different solid tumors; nevertheless, the elevated risk of hemorrhage incidents jeopardizes their uses. 51 The main objective of this study was to evaluate the effect of a heparin with low-anticoagulant activity (named as LABH), a derivative from pharmaceutical preparations of HBI, on tumor progression in mice. The effect of this derivative was compared with that of the gold standards HPI and enoxaparin (LMWH). The three types of heparins were SC administered to mice at the same dose in mass and not as anticoagulant potency (IU) since the effect we tested is not related to the action of the heparins on coagulation.

The administration of HPI delayed tumor progression but in parallel it had a toxic effect in the animals, as indicated by the increased mortality, some due to bleeding. In contrast, LABH had a similar effect in hindering tumor progression, but without such deleterious effects. These observations show that LABH has a wider therapeutic window than HPI, with beneficial pharmacological action and diminished adverse effects. Considering that UFHs are commonly administered IV, another challenge we overcome here was to ensure that LABH is satisfactorily absorbed after SC administration, which is an essential route for outpatient use over long periods of time.

Finally, our study is an example of combining the detailed structural analysis of heparin preparations with testing their pharmacological effects on both in vitro assays and specific animal experimental models. This is an approach to obtain more accurate information on the structure versus specific biological effects, which may lead to the development of new heparins with practical use in medicine.

## Notes

**What Is Known about This Topic?**

- Heparin has several pharmacological properties other than anticoagulant activity, especially for use as new oncogenic agent.
- However, the high anticoagulant potency of heparin makes the doses necessary to exert non-hemostatic effects unsafe due to an elevated bleeding risk.
- Development of new heparins with decreased anticoagulant activity could make the therapeutic use for treating non-thromboembolic diseases safer.

**What Does This Paper Add?**

- We demonstrate that a low-anticoagulant bovine heparin derivative is suitable for subcutaneous administration required for outpatient/long-term treatments.
- Even high subcutaneous doses of this derivative do not provoke bleeding.
- This derivative was able to delay the early progression of Lewis lung carcinoma, improve survival, and bring health benefits with minimal adverse effects.
- Such a low-anticoagulant heparin is a promising candidate for development as a new therapeutic agent for treatment of non-thromboembolic diseases.
